# Hyperprolactinemia and insulin resistance in drug naive patients with early onset first episode psychosis

**DOI:** 10.1186/s12888-018-1827-3

**Published:** 2018-08-01

**Authors:** Maria Giuseppina Petruzzelli, Mariella Margari, Antonia Peschechera, Concetta de Giambattista, Andrea De Giacomo, Emilia Matera, Francesco Margari

**Affiliations:** 1 Child Neuropsychiatry Unit, Department of Basic Medical Sciences, Neuroscience and Sense Organs, University of Bari “Aldo Moro”, Azienda Ospedaliero-Universitaria Policlinico di Bari, Piazza Giulio Cesare 11, 70124 Bari, Italy; 2 Psychiatry Unit , Department of Basic Medical Sciences, Neuroscience and Sense Organ, University of Bari “Aldo Moro”, Azienda Ospedaliero-Universitaria Policlinico di Bari, Piazza Giulio Cesare 11, 70124 Bari, Italy

**Keywords:** Prolactin regulation, Glucose tolerance, Neuroendocrine dysfunctions, Schizophrenia spectrum psychosis, Clinical high risk of psychosis, Adolescence, Stress

## Abstract

**Background:**

Hyperprolactinemia and glucose and lipid metabolism abnormalities are often found in patients with schizophrenia and are generally considered secondary to the use of antipsychotic drugs. More recent studies have shown these same neuroendocrine and metabolic abnormalities in antipsychotic naïve patients with first episode psychosis (FEP), rising the hypothesis that schizophrenia itself may be related to an abnormal regulation of prolactin secretion and to impaired glucose tolerance. The aim of this study was to compare prolactin levels, glycometabolism parameters and lipid profile between a sample of 31 drug-naive adolescents in the acute phase of FEP and a control group of 23 subjects at clinical high risk (CHR) of developing psychosis.

**Methods:**

The assessment involved anthropometric data (weight, height, BMI index, pubertal stage) and blood tests (levels of glucose, glycated hemoglobin, serum insulin, triglycerides, total and fractionated cholesterol, prolactin). Insulin resistance (IR) was calculated through the homeostatic model of assessment (HOMA-IR), assuming a cut-off point of 3.16 for adolescent population. FEP patients and CHR controls were compared by using Student’s *t*-distribution (*t*-test) for parametric data. *P* < 0.05 was considered significant.

**Results:**

Significant higher level of prolactin was found in FEP group than in CHR group (mean = 28.93 ± 27.16 vs 14.29 ± 7.86, *P* = 0.009), suggesting a condition of hyperprolactinemia (HPRL). Patients with FEP were more insulin resistant compared to patients at CHR, as assessed by HOMA-IR (mean = 3.07 ± 1.76 vs 2.11 ± 1.11, *P* = 0.043). Differences of fasting glucose (FEP = 4.82 ± 0.71, CHR = 4.35 ± 0.62, *P* = 0.016) and HbA1c (FEP = 25.86 ± 13.31, CHR = 33.00 ± 2.95, *P* = 0.013), were not clinically significant as the mean values were within normal range for both groups. No significant differences were found for lipid profile. A BMI value within the range of normal weight was found for both groups, with no significant differences.

**Conclusion:**

We suggested that HPRL, increase in HOMA-IR, and psychotic symptoms may be considered different manifestations of the acute onset of schizophrenia spectrum psychosis, with a common neurobiological vulnerability emerging since adolescence. The influence of age and gender on clinical manifestations of psychotic onset should be considered for early prevention and treatment of both schizophrenia spectrum psychosis and neuroendocrine-metabolic dysfunctions.

## Background

Hyperprolactinemia (HPRL) and glucose and lipid metabolism abnormalities are often found in patients with schizophrenia and are usually considered secondary to the use of antipsychotic drugs [1–4]. The production of prolactin (PRL) is inhibited by dopamine release in the hypothalamo-pituitary circuit and can be increased by blocking type 2 (D2) dopamine receptors [5]; therefore HPRL in patients with psychosis seems to be related mainly to the D2-receptors affinity of antipsychotic drugs [6]. Moreover, second generation antipsychotics (SGAs) have a marked propensity to induce increased levels of blood glucose, glycosylated hemoglobin and insulin, higher IR, alterations of blood lipid profiles, weight gain and sometimes frank diabetes mellitus (T2DM) [7–9]. The focus of these metabolic abnormalities is on the phenomena of hyperglycemia and IR, but the mechanism through which SGAs induce glucose metabolism disorders is not clearly understood and is likely multifactorial [3].

In more recent years it’s been purposed that HPRL and impaired glucose tolerance may be independent of antipsychotic treatment, at least in a subgroup of patients with psychotic disorders. Some authors have identified variations in PRL secretion in drug-free patients, suggesting that schizophrenia itself may be characterized by an abnormal regulation of PRL levels [5, 10–13]. Likewise some studies showed that antipsychotic naive patients with first episode psychosis (FEP) had increased prevalence of impaired glucose tolerance and were more insulin resistant than their healthy comparison subjects [14–16]. These findings lead to the hypothesis that schizophrenia spectrum psychosis is implicated in the pathophysiology of diabetes [15] although conflicting data on the risk of T2DM in patients with schizophrenia prior to antipsychotic treatment has been published [17, 18].

In this study we moved from the hypothesis that at least a subgroup of patients with schizophrenia spectrum psychosis may have a specific vulnerability for abnormalities of regulation of PRL secretion and impaired glucose tolerance, independent of antipsychotic treatment. We purposed that HPRL and abnormalities of glucose and lipid metabolism parameters may be related to the acute phase of psychosis. To verify this hypothesis we compared PRL levels, glycometabolism parameters and lipid profile between a sample of drug-naive adolescents in the acute phase of FEP and a control group of subjects at clinical high risk (CHR) of developing psychosis, age and sex matched.

## Method

### Subjects

The study sample included 31 patients of both sexes, aged between 8 and 18, consecutively referred over a three-years period, from 2014 to 2017, among the inpatients of the Child Neuropsychiatry Unit, Department of Basic Medical Sciences, Neurosciences and Sense Organs, University “Aldo Moro” of Bari, Italy. They were in the acute phase of their FEP, defined as the manifestation of delusion, hallucination and/or disorganization symptoms of less than 6 months’duration at the time of the assessment. Diagnoses of early onset first-episode schizophrenia spectrum psychosis (schizophrenia, schizophreniphorm disorder, schizoaffective disorder, psychosis not otherwise specified) were made in accordance to Diagnostic and Statistical Manual for Mental Disorders-fifth edition (DSM-5) criteria, on the basis of clinical evaluations and supported by using of the Italian version of the Kiddie-Schedule for Affective Disorders and Schizophrenia-Present and Lifetime Version, (K-SADS-PL), a semi-structured diagnostic interview [19]. All patients were clinically assessed within the first 72 h after their admission to the Child Neuropsychiatry Unit, by the validated Italian version of the Positive and Negative Syndrome Scale (PANSS), a standardized instrument including 30 items on a seven point scale to assess positive, negative and general symptoms [20]. The assessment was performed following a semi-structured interview to ensure that all content domains were covered during the evaluation session; the scores were assigned according to PANSS rating criteria. The control sample consisted of 23 patients, referred to the same Child Neuropsychiatry Unit, age and sex matched, defined as at CHR state of psychosis on the basis of the ultra-high risk (UHR) and the basic symptoms criteria, as indicated by the European Psychiatric Association (EPA) guidance on the early detection of clinical high risk states of psychosis [21]. The UHR criteria, defined as the presence of al least one of attenuated psychotic symptoms (APS), brief limited intermittent psychotic symptoms (BLIPS) or genetic risk and functional decline (GRFD), were assessed with the Italian version of Comprehensive Assessment of At Risk Mental State (CAARMS), a semi-structured interview developed to measure a range of sub-threshold symptoms associated with the prodromal phase of psychotic disorders [22]. The basic symptoms criteria were assessed with the Italian version of Schizophrenia Proness Instrument - Child & Youth version (SPI-CY), a semi-structured interview developed to assess basic symptoms in 8 to 18 year olds [23]. Two experienced child and adolescent psychiatrists from the research group, trained in the use of the above mentioned instruments, interviewed parents and patients; all the evaluations were discussed in regular reliability meetings, supervised by the senior researcher. Body weight (Kg) and height (m) of each study participant were measured and their body mass index (BMI, kg/m^2^) was calculated. The pubertal stage was evaluated using visual inspection and palpation, according to Tanner classification for breast development in girls and for genitalia in boys. Exclusion criteria were age < 8 years or > 18 years, antipsychotics intake before the enrolment, evidence form medical history, physical examination, laboratory and instrumental findings that psychotic symptoms were substance induced or due to another medical condition, clinical and instrumental evidences of medical causes of HPRL, no history of diabetes or other serious medical condition associated with glucose intolerance or IR. After providing complete description about the study, we obtained written informed consent from the parents of all subjects. The study was approved by the Ethical Committee of the Hospital Consortium Policlinico of Bari, Italy.

### Biochemical analyses

Peripheral blood samples from all partecipants were collected between 7 and 8 AM, following an overnight fast. Serum PRL levels were measured by Advia Centaur XP Immunoassay System (Siemens, Erlangen, Germany). HPRL was defined as a PRL level more than 20 ng/ml in males and more than 25 ng/ml in females. Serum glucose was estimated by enzymatic method; levels between 4 and 5,5 mmol/L were considered normal for both sexes. Glycated hemoglobin (HbA1c) was determined by High Performance Liquid Chromatography; levels between 20 and 42 mmol/L were considered normal for both sexes. Serum insulin was determined by chemioluminescence. Hyperinsulinemia was defined as a value more than 16 microUI/mL, for both male and female patients. HOMA-IR was calculated through the homeostatic model of assessment as the product of the fasting plasma insulin level (μU/mL) and the fasting plasma glucose level (mmol/L), divided by 22.5; a HOMA value > 3.16 was considered indicative of IR [24, 25]. Total cholesterol (mg/dl) levels were determined by a traceable IFCC standardized method. Levels of 156 ± 4,9 (mg/dl) for male and 173 ± 4,5 (mg/dl) for female were considered normal. High-density lipoprotein cholesterol (HDLc) was measured by clearance assay. Levels between 39 ± 1,4 mg/dl for male and 45 ± 1,2 for female were considered normal. Fasting plasma levels of low-density lipoprotein cholesterol (LDLc) were calculated by using of Friedewald et al. (1972). LDLc levels of 101 ± 4,6 mg/dl for male and 108 ± 3,9 mg/dl were considered normal.

Triglycerides levels were determined by a traceable IFCC standardized method. Levels within the range 22–138 mg/dl were considered normal for both sexes.

### Statistical analyses

Data analysis was performed by using SPSS 13.0 software. Data were expressed as mean ± standard deviation (S.D.) for continuous variables; qualitative variables were expressed as percentage. Demographic and clinical characteristics were compared using χ^2^ test or Mann-Withney U test as appropriate. Clinical observed variables between groups were compared by using Student’s *t*-distribution (*t*-test) for parametric data. *P* < 0.05 was considered significant.

## Results

The demographic and clinical features of the two samples of study were resumed in Table 1. Patients with FEP did not differ from patients at CRH with respect to age, gender and Tanner stage. Table 2 described data on PRL levels, glycometabolism parameters, lipid profile and BMI. The FEP group showed a significant higher level of PRL than the CRH group (*P* = 0.009), with a mean value corresponding to a condition of HPRL (28.93 ± 27.16). When we compared glucose metabolism parameters we found significant differences of fasting glucose (*P* = 0.016) and HbA1c (*P* = 0.013) between FEP and CRH. These data were not clinically significant as the mean values for both groups were within normal range. We found a significant difference of mean value of HOMA-IR between FEP and CRH (*P* = 0.043), with a value of 3.07 ± 1.76 close to the reported cut off of IR. No significant differences were found for parameters of lipid metabolism, with values within normal range. A BMI value within normal range was found in both groups, with no significant statistical difference between FEP and CRH.

**Table 1:** Sociodemographic and clinical characteristics in FEP and CHR group

	FEP (*n* = 31)	CHR (*n* = 23)	*P* value
Age in years ^a^(mean ± S.D.)	15.8(±1.30)	14(±1.41)	0.064
Gender ^b^			0.56
Male n(%)	10(32.26%)	8 (34.78%)	
Female n(%)	21 (67.74%)	15 (65.22%)	
Tanner stage^b^ n(%)			0.68
I	–	1 (4.35%)	
II	–	3 (13.04%)	
III	6 (19.35%)	8 (34.78%)	
IV	13 (41.93%)	6 (26.08%)	
V	12 (38.72%)	5 (21.75%)	
PANSS total score(mean ± S.D.)	92(± 8.54)	–	
At risk criterion			
UHR n(%)	–	16 (69.57%)	
Coper/COGDIS n(%)	–	7 (30.43%)	

^a^ Mann-Whitney U test

^b^χ^2^ test

**Table 2:** Comparison between FEP and CHR of prolactin values, glucose metabolic parameters, lipid profile and BMI

	FEP (n = 31)	CHR (n = 23)	*P* values
Prolactin (ng/ml)	28.93 (27.16)	14.29 (7.86)	**0.009**
Glucose metabolic parameters			
Fasting glucose (mmol/l)	4.82 (0.71)	4.35 (0.62)	**0.016**
HbA1c (mmol/mol)	25.86 (13.31)	33.00 (2.95)	**0.013**
Fasting insulin (μUI/ml)	14.01 (6.88)	11.08(4.67)	0.098
HOMA-IR	3.07 (1.76)	2.11 (1.11)	**0.043**
Lipid profile			
Total cholesterol (mg/dl)	137.23 (22.30)	150.56 (25.27)	0.051
HDL-cholesterol (mg/dl)	54.96 (12.96)	53.18 (12.68)	0.628
LDL-cholesterol (mg/dl)	71.6 0(18.89)	79.54 (19.59)	0.156
Tryglicerides (mg/dl)	58.83 (19.14)	91.95 (19.44)	0.156
BMI (kg/m^2^)	21.13(2.80)	21.50 (4.07)	0.731

Values are shown as mean (SD). Bold font is indicative of P < 0.05

## Discussion

The main findings of this study were significantly higher values of PRL and HOMA-IR in the sample of early onset drug-naive FEP when compared with subjects at CHR of developing psychosis. These data suggested that dysfunctions of regulation of PRL serum levels and abnormalities of glucose metabolism parameters could be related to acute phase of FEP more than to a clinical risk of psychotic illness, excluding the iatrogenic effects of antipsychotic drugs.

Some previous studies on patients with adult onset FEP showed that HPRL may be a condition pre-existing the introduction of antipsychotic drugs, at least in a subgroup of cases [10–13]. In addition genetic studies suggested that some patients with schizophrenia may have a predisposition to HPRL consisting in functional − 1449 g/t polymorphism of the PRL gene [26, 27]. In a review on the literature of the relationship between PRL and schizophrenia Rajkumar R.P. recommended a reappraisal of the role of prolactin in the various stage of schizophrenia, particularly with regard to its onset [5]. At this purpose an important concern involves the relationship between stress, psychosis onset and increased release of PRL. As is known acute stress leads to an increased serum level of PRL [28] and, in addition to it, patients with FEP report more stressful life events and perceived stress when compared to healthy subjects [29]. An increased level of PRL triggers dopamine release by a feedback mechanism, therefore it is reasonable to infer that patients with schizophrenia may have an exaggerated PRL response to stress mediated by a dysfunction in dopaminergic transmission [5]. Moreover, it has been hypothesized that in early psychosis a stress related hormonal dysregulation leads to a greater activation of the hypothalamic-pituitary-adrenal axis (HPA) with an increase in the number and size of corticotroph cells [30, 31]. The pituitary gland is a dynamic structure changing in response to different conditions. Nordholm et al. published a systematic review and meta-analysis on pituitary gland volume showing that the onset of psychosis, more than the high-risk state, is associated with an enlargement of the pituitary gland and that this is independent of antipsychotics [30]. On the other hand, pituitary size may be influenced by age and gender [32], so that subjects in juvenile age (puberal and post-puberal stage) and female in gender may be more easy to have an increase of pituitary gland volume and activation during the early stage of psychosis [32, 33]. All these evidences give more consistency to our finding of HPRL in acute phase of FEP more than in CHR subjects, so we suggest that neurobiological abnormalities related to the acute phase of full emergence of psychotic symptoms in adolescent patients may be involved in dysregulation of PRL secretion. Considering the development and sexual dimorphism of the pituitary gland, further studies could be useful to examine the relevance of age and gender differences on early onset psychiatric disorders in which pituitary dysfunctions has been implicated. In addition, the early identification of HPRL in drug naïve adolescents with FEP may be useful in clinical setting to guide treatment decisions and to manage antipsychotic drugs over the time.

A complex and multifactorial relationship it’s been purposed also to explain the risk of diabetes in psychotic disorders [2]. In a recent meta analysis examining data of glucose tolerance, insulin and insulin resistance from drug naïve adult patients with non affective psychosis (mean age of 28.7) Greenhalgh et al. purposed that an increased risk of diabetes may be apparent in acute phase of psychosis prior to antipsychotic use. The authors found that at the time of first clinical contact for psychosis, patients have a slight increase in fasting glucose, which most of them maintain in the normal range, despite a small increase in IR, by secreting additional insulin [34]. According to these evidences, we found a higher fasting glucose in the FEP group then in the CHR group, with no clinical significance, as the mean values for both groups were within normal range. The mean value of HbA1c, reflecting the average plasma glucose, was within normal range for both groups too, so we can assume that the statistical difference (HbA1c was higher in the CHR group than in the FEP group) was not of clinical significance. Actually, some previous studies showed that determination of fasting plasma glucose or HbA1c is not very useful in the screening of impaired glucose tolerance [35, 36]. Conversly Garcia-Rizo et al. have recently suggested that at the onset of psychosis HOMA-IR may be considered a more useful predictor of cardiometabolic risk than other metabolic syndrome criteria [37]. The HOMA-IR is a proxy estimate of IR, based upon the relationship between fasting glucose and insulin levels, with higher value of HOMA-IR representing more severe IR [38]. Several authors have recently purposed a definition of HOMA-IR values across the age continuum from childhood to adolescence, identifying IR with more specificity and sensibility [39, 40]. Actually, there is no consensus regarding the reference value of HOMA-IR for the diagnosis of IR in the pediatric age group and several cut off points have been reported in the literature according to age, gender, pubertal status and BMI [38, 41, 42]. The most used cut off value is 3.16 for obese children, considering that in obese young individuals IR may exceed physiological values especially at the time of puberty [24, 25, 41]. Different population-based studies in the world on normal weight healthy children and adolescents found that a HOMA-IR ≥ 2.6 was associated with a greater cardiovascular and metabolic risk [38, 43, 44]. According to these data we can assume that the mean value of HOMA-IR of 3.07 we found in the FEP group was indicative of IR, since the BMI of these patients was in the range of normal weight. So we suggest that since adolescence, as up to now purposed for adulthood [34], a higher risk of impaired glucose homeostasis, assessed by an increase in HOMA-IR, may be evident in the acute phase of FEP, also in absence of other risk factors as overweight and independently by antipsychotic intake. This is of substantial clinical importance to identify adolescent patients with FEP at increase cardiovascular risk, in order to implement preventive strategies and optimize therapies to reduce the burden of weight gain relate to antipsychotic drugs.

We reserved one last comment to the hypothesis of a deep relationship between HPRL and IR in schizophrenia spectrum psychosis. In a 2016 review Gragnoli et al. suggested that in schizophrenic patients neuroendocrine dysfunctions involving dopamine-prolactin pathway might contribute to both diabetes and schizophrenia [45]. This hypothesis recalls the multifunctional role of PRL that, beside the lactogenic activity, has different functions broadly classified as reproductive, metabolic, osmoregulatory and immunoregulatory [46]. As “metabolic hormon” increasing evidence showed that PRL has different effects on glucose metabolism and may be involved in the manifestation of IR [47, 48]. On the other hand we know that IR is not an endocrine disorder per se but more a disorder of several systems that appears in many inflammatory conditions with an activated immune/repair system and/or in different conditions with increased mental activation via stress axes [49, 50]. So Gragnoli et al. purposed that PRL and/or PRLreceptor gene may carry risk variants associated with schizophrenia, T2DM and/or their clinical association [45]. This hypothesis is in line with a contemporary conceptual model of schizophrenia as a complex disorder with several manifestations outside the brain, so that some “non-psychiatric” abnormalities, traditionally considered as “comorbid” conditions, actually appear as integral parts of the illness, sharing etiopathophisiological factors [51] (Brian Kirkpatrick 2015). In this framework we can suggest that our finding of co-occurring HPRL, increase in HOMA-IR and psychotic symptoms may be considered different manifestations of the acute phase of psychotic onset with a common neurobiological vulnerability.

Some limitations need to be considered. Certainly the small sample size of this study limited the statistical power of the results and made difficult to evaluate the effect of age, sex, pubertal stage, phase of disease on neuroendocrine and metabolic parameters. We must consider, in addition, that this is a cross-sectional study. Further replications trough longitudinal studies including larger samples would be needed to a better understanding of the relationship between schizophrenia spectrum psychosis, regulation of prolactin secretion and IR, according to different age of onset and different stage of disease.

## Conclusion

In conclusion this study showed HPRL and an increase in HOMA-IR in the acute phase of early onset FEP but not in CHR of developing psychosis, rising the needs to look at schizophrenia spectrum psychosis as a complex and multifactorial clinical condition in which neuroendocrine and metabolic abnormalities may be considered integral parts of a wide spectrum of neurobiological dysfunctions. Genetic predisposition, immune system activation, stress related factors are most likely the common mediators explaining the co-occurrence of neuroendocrine and metabolic dysfunctions in acute phases of psychosis and will become important directions for further research. A better understanding of the influence of age and gender on clinical manifestations of psychotic onset may be useful for early prevention and treatment of both schizophrenia spectrum psychosis and neuroendocrine-metabolic dysfunctions.

## Abbreviations

APS: Attenuated Psychotic Symptom
BLIPS: Brief Limited Intermittent Psychotic Symptom
BMI: Body Mass Index
CAARMS: Comprehensive Assessment of At Risk Mental States
CHR: Clinical High Risk
D2: Dopamine 2
DSM-5: Diagnostic and Statistical Manual for Mental Disorders-fifth edition
EPA: European Psychiatric Association
FEP: First Episode Psychosis
GRFD: Genetik Risk and Functional Decline
HbA1c: Glycated Hemoglobin
HDLc: High-density Lipoprotein cholesterol
HOMA-IR: Homeostatic Model of Assessment-Insuline Resistance
HPA: Hypothalamic-Pituitary-Adrenal axis
HPRL: Hyperprolactinemia
IR: Insuline Resistance
LDLc: Low Density Lipoprotein cholesterol
PANSS: Positive and Negative Syndrome Scale
PRL: prolactine
SD: Standard Deviation
SGAs: second generation antipsychotics
SPI-CY: Schizophrenia Proness Instrument, Child & Youth Version
T2DM: Type 2 Diabetes Mellitus
UHR: Ultra-High Risk
